# Integrating presbyopia services into community and primary health care

**Published:** 2026-03-12

**Authors:** Olufunke Fasawe, Amit Gupta, Julius Mbeya, Abraham Zerihun Megentta, Oteri Okolo, Patricia Elaine Freels, Elanor Watts

**Affiliations:** 1 Vice President, Integration and Country Director, Nigeria: Clinton Health Access Initiative, Abuja, Nigeria.; 2 COO: The/Nudge Institute, Bangalore, India.; 3 Co-CEO: Lwala Community Alliance, Rongo, Kenya.; 4 Country Director, Ethiopia: Last Mile Health. Addis Ababa, Ethiopia.; 5 National Eye Care Coordinator and Director: National Eye Health Programme, Nigeria.; 6 Communications: Livelihood Impact Fund, Istanbul, Turkey.; 7 Research Consultant: Peek Vision, Technical Advisor: LIF Eyeglasses Initiative, and Ophthalmology Resident: NHS Scotland, Glasgow, UK.

**Community and primary health workers are a helpful gateway to near-vision care.**

**Figure:**
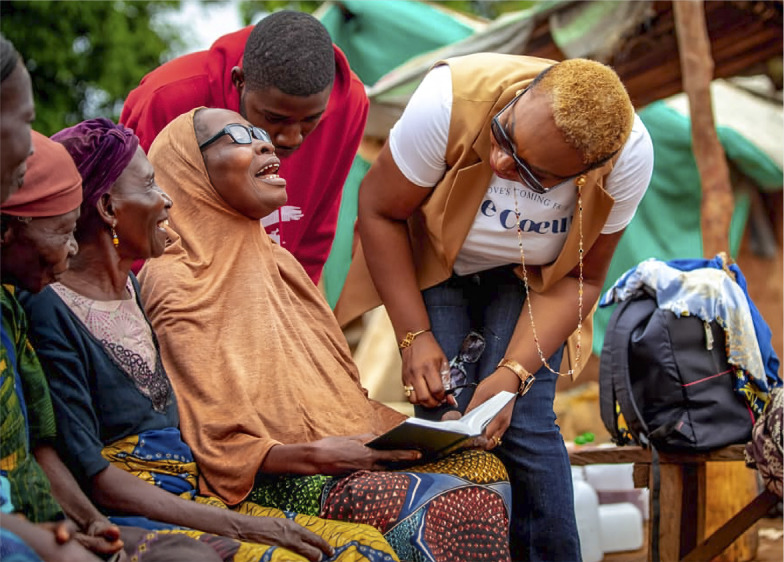
A woman is delighted that she can read her religious text after receiving a pair of near-vision spectacles. NIGERIA

© NESHP/CHAI CC BY-NC-SA 4.0

The World Health Organization (WHO) states that community health workers and primary health workers can be safely trained to screen for presbyopia and to distribute ready-made near-vision spectacles (see article on pp. 4-5).

Globally, approximately 4.7 million community health workers in around 100 countries give people access to life-saving information, testing, and medications.^1^ They are trained to travel to where people live and work, which allows them to provide care to communities in remote or hard-to-reach areas, also known as the ‘last mile.’ In contrast, primary health workers are normally based at local health centres, where they provide essential health care services to the people who travel to them from nearby communities.

WHO recommends that both community and primary health workers be included in an integrated, competency-based refractive error team (Figure 1).^2^ Community health workers can be trained to educate communities about presbyopia, carry out near-vision screening, distribute near-vision spectacles, and refer those with other eye health needs to the relevant service or level in the health system (Figure 1). Primary health care workers can conduct distance and near visual acuity tests to detect people needing referrals and provide ready-made near-vision spectacles for those who only need near-vision correction. This approach improves access to eye care, and it frees other members of the refractive error team to focus on patients with more complex eye conditions.

As trusted community members, community and primary health workers are a helpful gateway to eye health care. In countries where there is little or no awareness of presbyopia, it is helpful for a trusted person within the community to provide people with their first pair of spectacles.

Health care workers themselves also need access to near-vision spectacles,^3,4^ as there are many skills which require near vision, such as recording or reviewing data on patient health records, providing immunisations, reading blood pressure monitors and thermometers, and reading medication labels. Some tasks, such as suturing, cannot be done safely without good near vision.

Several countries have already started projects that support community and primary health workers to provide eye screening for presbyopia and distribute near-vision spectacles.

## Ethiopia

In Ethiopia, community health workers, known nationally as health extension workers, are being trained to provide eye screening and near-vision spectacles as part of a new non-communicable disease training package. Following a successful pilot in 12 districts, in which over 650 health extension workers were trained, the training is being scaled nationally. The distribution of near-vision spectacles is currently being expanded to reach hundreds of thousands more patients in areas that are often the hardest to reach.

**Figure 1:**
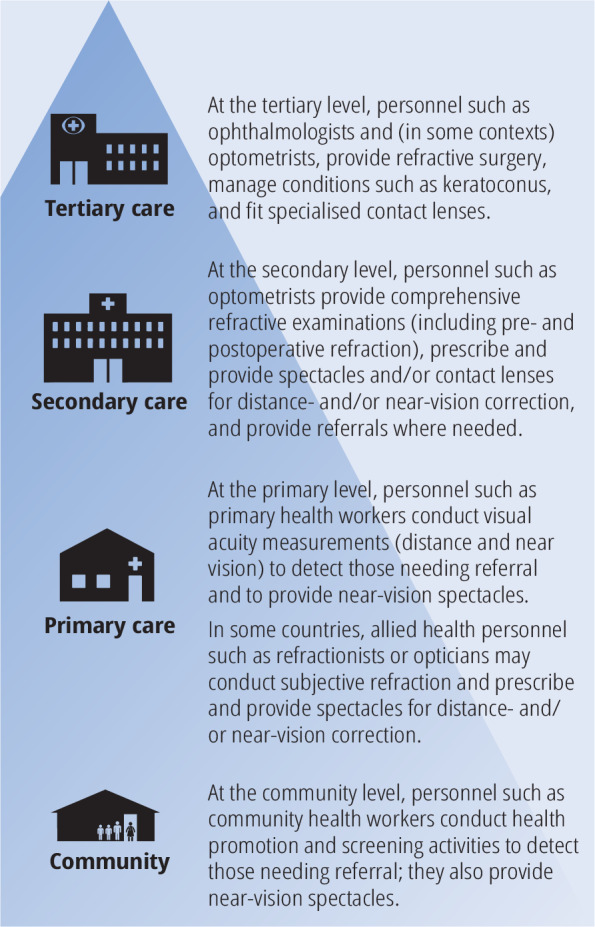
Refractive error personnel integrated across all levels of the health system.

Source: WHO, Competency-based refractive error teams.^2^

**Figure:**
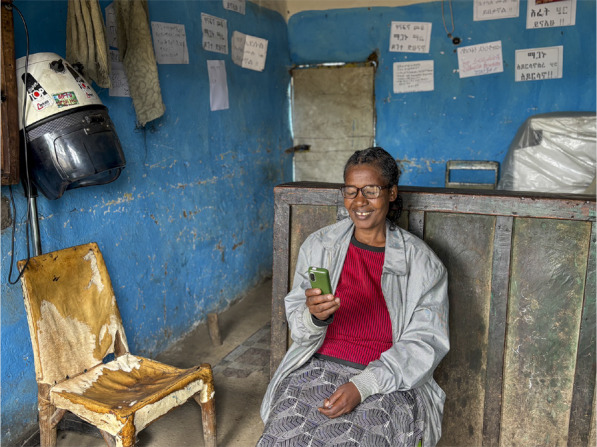
A woman running a small business can use her cellphone after receiving near-vision spectacles. ETHIOPIA

© LAST MILE HEALTH, ETHIOPIA CC BY-NC-SA 4.0

*“The key thing has been integration. This is not a standalone training, it's integrated with several other components, and this has multiple benefits. One is efficiency. You're not calling on health extension workers only to tell them about presbyopia, but also non-communicable diseases overall.”*

- Abraham Zerihun Megentta, Country Director, Ethiopia for Last Mile Health

## Kenya

Lwala is a Kenyan-led organisation training and supporting community health workers to dispense near-vision spectacles. With the support of the Livelihood Impact Fund, Lwala led a successful pilot project in Migori county, during which 280 primary health care workers and 269 community health worker supervisors were trained to screen for presbyopia. In turn, they trained an additional 3,214 community health workers. As a result of this project, a total of 53,000 people in the county were screened and nearly 50,000 near-vision spectacles were distributed. Lwala will now be scaling up this work in additional counties within Kenya.

## India

India has 1 million Accredited Social Health Activists (ASHAs) and approximately 178,000 functional Ayush Arogya Mandirs (primary health workers) who are embedded in villages in India. Integrating presbyopia screening and spectacle distribution into their workflows is low cost and sustainable. In addition, India's 9 million self-help groups, representing over 100 million members, provide a powerful grassroots network. The/Nudge Institute is piloting near-vision spectacle distribution through these health and development channels to extend access to near-vision care across the country (see the article on p. 13 in this issue).

## Nigeria

The government of Nigeria has launched a new presidential initiative: the Effective Spectacle Coverage Initiative Nigeria. The project is led by Dr Oteri Okolo, National Coordinator of the National Eye, Ear and Sensory Functions Health Programme of Nigeria, and is implemented in partnership with the Livelihood Impact Fund, Clinton Health Access Initiative Nigeria, Christian Health Association of Nigeria, Founders Pledge, and RestoringVision. It aims to reach 5 million people with near-vision spectacles through primary health centres and community outreach efforts. In the last year, over 1.3 million Nigerians received free reading spectacles, and approximately 1.5 million people across 16 states received vision screening. Two-thirds of these beneficiaries received their first-ever pair of spectacles, reaching those historically excluded from the eye care sector. The programme has trained over 2,200 primary health care workers, with plans to expand in its second year. Integrating primary eye care into the primary health care system in this way means that - once the initiative ends - the primary health care system can remain a source of near-vision spectacles, thereby improving the initiative's long-term sustainability.

Fortunately, ready-made near-vision spectacles are safe in adults: an incorrect dioptre can result in eye strain or a headache, but this prompts the wearer to remove them and will not cause permanent damage.

Given both the safety and benefits of near-vision spectacles, we hope that - by integrating near-vision testing and spectacle provision in the work of community and primary health care workers - it will be possible to reach millions of people every year. Moreover, by giving health workers the near-vision spectacles they need, we aim to improve the accuracy and effectiveness of the vital work they do.

**Training for community and primary health workers**

There are various approaches to training community and primary health care workers in ways that are both scalable and highly effective.

Training sessions range from a few hours to multiple days. For example:

- Clinton Health Access Initiative in Nigeria found that a 1-2 hour training session on presbyopia and near-vision spectacles, as part of the National Primary Eye Care Training Manual (the local version of the WHO AFRO Primary Eye Care package) was sufficient, particularly when complemented by supportive supervision.
- RestoringVision created an eight-minute training video (bit.ly/RVnvtraining) on the provision of spectacles. In many programmes, refresher training is also offered.
- WHO provides an open-access learning course, “Learning on TAP” (Training in Assistive Products) for primary health care and community workers specifically for vision screening. This course takes 60 to 90 minutes to complete and aims to get health care workers up to speed and ready for in-person practice in the workforce. See bit.ly/WHOtapVision
- Last Mile Health in Ethiopia embedded near-vision training directly into the national non-communicable disease training module for community health workers, in partnership with the Ethiopia Ministry of Health. Community health workers there showed an impressive rate of skill uptake, with 80% passing the post-training assessment compared to 10% pre-training. See more here: bit.ly/LastMileH
- Digital tools developed by Peek Vision (peekvision.org) can walk a community or primary health worker through the process of testing near visual acuity and then providing ready-made near-vision spectacles, starting with an appropriate dioptre based on the person's age and near acuity, and then supporting a trial of different powers to find the preferred, effective power.
- Simple posters (included in this issue), placed in primary health care settings, can remind health care workers to test for presbyopia and provide near-vision spectacles.
